# Guiding principles for establishing and strengthening environmental microbiology laboratories for public health in resource-limited settings

**DOI:** 10.1371/journal.pwat.0000543

**Published:** 2026-05-04

**Authors:** Jennifer Murphy, Daniele Lantagne, Jonathon Gass, Alexandra Kossik, Javier Gallard-Góngora, Molly Cantrell, Dan Deere, Manise Pierre, Vincent Hill

**Affiliations:** 1 Waterborne Disease Prevention Branch, Centers for Disease Control and Prevention, Atlanta, Georgia, United States of America; 2 Feinstein International Center, Friedman School of Nutrition, Tufts University, Medford, Massachusetts, United States of America; 3 Department of Public Health and Community Medicine, School of Medicine, Tufts University, Boston, Massachusetts, United States of America; 4 Water Futures, Sydney, New South Wales, Australia; 5 American Society for Microbiology, Washington, District of Columbia, United States of America

Environmental microbiology (EM) laboratories use specialized techniques to test environmental samples for microorganisms and physicochemical parameters across a range of applications. In the context of infectious disease prevention and control, EM laboratories routinely test water to identify contamination prior to waterborne disease outbreaks as part of preparedness efforts, and provide critical data during outbreaks to inform timely public health response in alignment with the 7–1-7 framework [1]. EM laboratories also support public health by tracking community-level infection trends through wastewater and environmental surveillance [2].

While the need to improve clinical laboratory capacity in resource-limited settings has been formally recognized for combatting existing and emerging health threats [3], advancing EM laboratory capacity has received limited attention. Despite the clear public health rationale for enhancing this capacity, implementation is difficult. Laboratories in resource-limited settings typically encounter challenges, including minimal infrastructure, inadequate access to reagents and equipment, and trained personnel shortages [4]. In addition, some laboratories may face pressure from national public health institutions, donors, or academic partners to implement EM methodologies without sufficient technical or financial support. These limitations can compromise quality, confidence in results, and program sustainability.

Strengthening EM laboratory capacity encompasses establishing infrastructure, optimizing operations, and ensuring timely communication of data to inform response and improve public health outcomes. Based on our collective experience collaborating with laboratories in resource-limited settings, we propose six guiding principles for public health officials and laboratory directors in countries seeking to establish and strengthen EM capacity, and for partner organizations assisting in these efforts (Fig 1):

## 1. Leverage local expertise and capacities

Understanding the local testing landscape prevents duplication and ensures services address public health gaps. Environmental testing often involves multiple disciplines and governmental jurisdictions (e.g., Ministries of Health, Water, and Environment) and can serve different functions (e.g., regulatory, response, research). Surveying existing in-country EM laboratories can reveal how current testing contributes to public health, uncover gaps in data collection, and highlight areas for improvement. Additionally, mapping clinical, food, and other laboratories is important, as they can provide access to essential resources (e.g., autoclaves, preparation areas, distillers) and advanced testing capabilities (e.g., polymerase chain reaction, sequencing) that may be infeasible for many EM labs to fund independently. It is also vital to identify EM champions within the country who understand the value of environmental data and can mobilize resources, foster collaboration, and maintain momentum for advancing EM initiatives.

## 2. Establish tiered EM laboratory infrastructure

Microbiological testing is sometimes conducted at sampling locations, but testing within a laboratory provides controlled conditions, more reliable data, and enhanced biosafety. Environmental samples are ideally processed in laboratory spaces separate from clinical testing to prevent diagnostic workflow contamination. Following the model of the Global Polio Laboratory Network [5], a tiered system of EM laboratories can improve access to timely, high-quality data to strengthen public health response while optimizing the use of limited resources. A network of geographically distributed sub-national EM laboratories that focus on basic water quality testing requires limited infrastructure and equipment and can be located near sample collection sites to meet recommended sample storage times (e.g., < 6 h for microbiological analysis [6]). Fewer EM laboratories are needed for specialized testing, such as pathogen detection or testing of additional matrices; these could be located in large urban centers, which are more likely to offer enhanced infrastructure, improved reagent supply chains, and access to expert personnel.

## 3. Optimize testing strategies

Thoughtful selection of EM testing strategies can yield data that support rapid public health response and ensure optimal use of scarce resources. At the local level, focusing on core screening tests for water quality, including culture-based detection of fecal indicator bacteria and measurement of basic physicochemical parameters, allows for relatively low-cost, high-volume testing that can provide rapid data for remediation or closure of contaminated sources. When additional information is needed, samples may be analyzed in advanced laboratories using complex and costly tests, such as pathogen culture or molecular assays. However, detecting fecal contamination or the absence of chlorine is often sufficient to inform public health action, and detailed pathogen identification may not provide additional actional information while also increasing time and resource demands. To create a sustainable funding model, laboratories could offer select paid testing services to commercial sectors and explore public-private and academic partnerships to support advanced testing without compromising core testing.

## 4. Prioritize laboratory quality and safety

A comprehensive quality management system (QMS) and laboratory safety are essential for ensuring reliable results, protecting staff and community health, and fostering trust with stakeholders. Resource-limited EM laboratories often encounter challenges such as reporting data without quality metrics (e.g., positive and reagent controls) and operating without biosafety controls (e.g., personal protective equipment, handwashing supplies) due to limited financial support. Strong QMS foundations – including inventory management, standard operating procedures, biosafety protocols, and accreditation—as well as standardized data management procedures require dedicated funding and are an important consideration alongside broader laboratory funding commitments.

## 5. Foster professional development

A laboratory’s greatest asset is its workforce. In resource-limited settings, low salaries contribute to high staff turnover rates. This, coupled with an increase in technological innovations available in the marketplace, can lead to a reliance on automation. However, skilled scientists remain indispensable for identifying issues or anomalies that guide investigations and improve methodologies [7]. Focusing on professional development across disciplines, continuing education, and career advancement opportunities helps retain talent and ensures staff can meet evolving challenges. Offering opportunities for collaboration with interdisciplinary experts and participation in mentoring programs facilitates rigorous, integrated, and consistent testing and data interpretation.

## 6. Ensure data-driven, timely public health action

Timely communication of EM laboratory data through clearly defined reporting chains is essential to inform risk mitigation in outbreak response and guide actions to improve health outcomes. For example, strategically selecting sampling locations, target microorganisms, and testing frequencies during outbreaks and expeditiously sharing results with relevant stakeholders such as water utilities facilitates prompt public health interventions and supports effective use of limited resources. Adoption of data management tools and cloud-based platforms can streamline laboratory processes, help summarize data quickly, and ensure timely results dissemination to enable swift public health action.

The findings and conclusions in this manuscript are those of the authors and do not necessarily represent the official position of the U.S. Centers for Disease Control and Prevention.

## Figures and Tables

**Fig 1.**
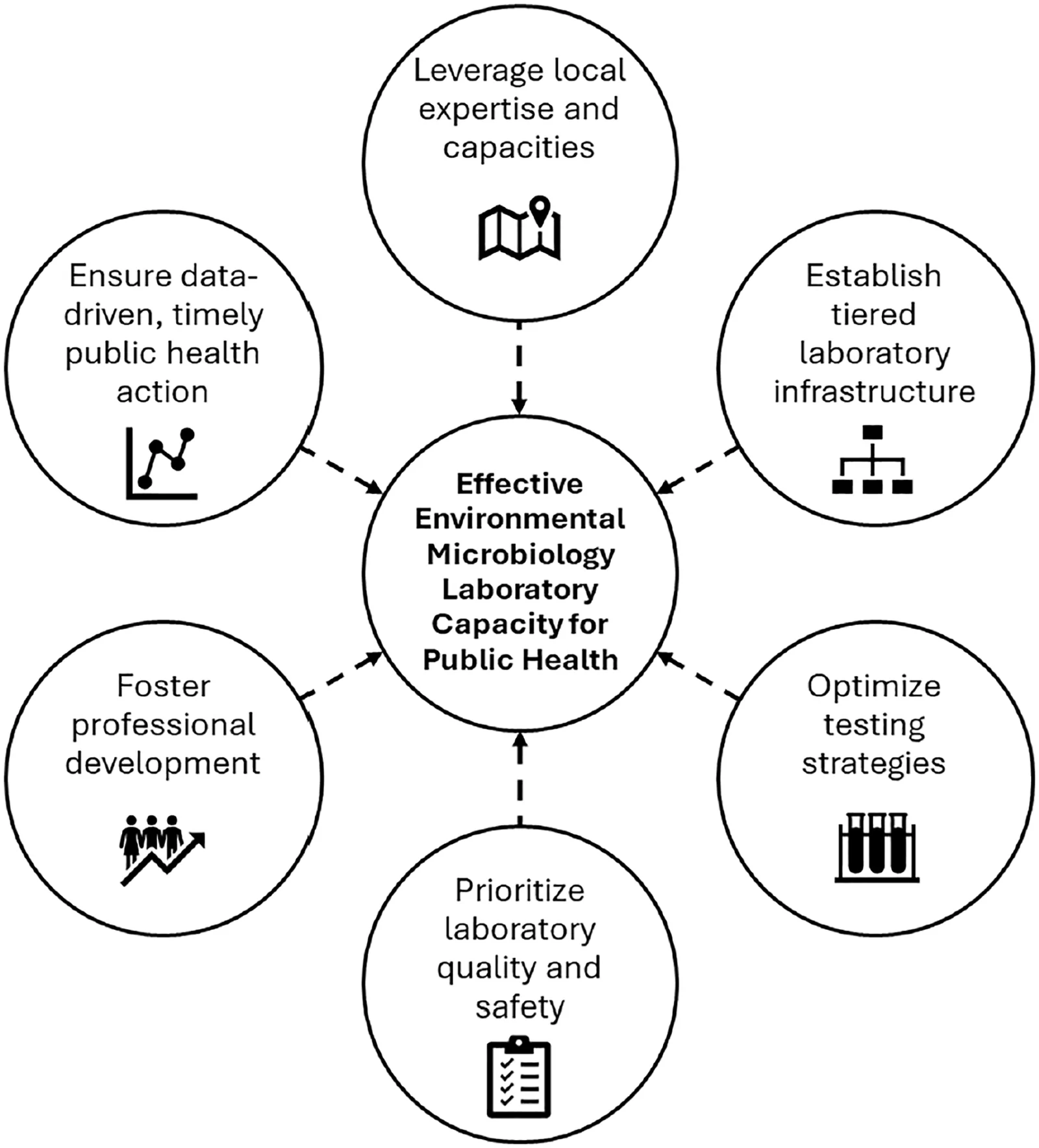
Six guiding principles for public health officials, laboratory directors, and partner organizations working to build and strengthen environmental microbiology capacity in resource-limited settings.
